# Supplementary material for: Collaborative intelligence in AI: Evaluating the performance of a council of AIs on the USMLE
Source: PLOS Digit Health. 2025 Oct 9;4(10):e0000787. doi: 10.1371/journal.pdig.0000787 (PMC12510544; doi:10.1371/journal.pdig.0000787)
Supplement: S2 File — (ZIP) [file pdig.0000787.s002.zip › S2 Data Raw data files containing unprocessed question inputs and LLM outputs including transcripts of deliberations/STEP3_Question-8-1_Type-MC-NJ.docx]

**Facilitator** **[Round 1]:** A 12-year-old boy with sickle cell disease and type 1 diabetes mellitus is brought to the office by his mother for an initial visit. The family recently moved to the area. Type 1 diabetes mellitus was diagnosed in the patient 6 years ago. Since that time, he has been treated with insulin and dietary management. His insulin regimen has not changed during the past year; however, his mother says he has been only marginally compliant with his insulin and dietary regimens. His diabetic diary shows home fingerstick blood glucose concentrations ranging from 140–200 mg/dL during the past 3 months. He admits to checking his glucose concentrations infrequently. Measurement of hemoglobin A1c obtained last week was 5.4%. The patient's vital signs are temperature 36.8°C (98.2°F), pulse 72/min, respirations 24/min, and blood pressure 110/64 mm Hg. Physical examination shows no abnormalities. Which of the following is the most likely explanation for the discrepancy between the patient's home fingerstick blood glucose concentrations and his hemoglobin A1c? (A) He has iron deficiency anemia (B) His daily glucose control is better than recorded (C) His glucometer is reading falsely high and should be replaced (D) His hemoglobin A1c is likely a result of laboratory error and should be repeated (E) His sickle cell disease is affecting his hemoglobin A1c

**AI_A:** RESTATING THE QUESTION: A 12-year-old boy with both type 1 diabetes and sickle cell disease, despite showing evidence of inconsistent insulin and diet adherence, exhibits aberrant hemoglobin A1c (HbA1c) and blood glucose readings. The patient appears to have surprisingly high blood glucose readings (on his home glucometer ranging between 140-200 mg/dL), while his recorded HbA1c value of 5.4% suggests good long-term glycemic control. The query here is seeking to identify the reason for the dissimilar readings between these two measures of glycemic control. PLANNING: Several assumptions could account for this discrepancy. It could be a result of anemia, better glucose control, problem with the glucometer, lab error with the HbA1c or an influence of sickle cell disease. Evaluating each of these assumptions, the boy's existing sickle cell disease prompts us to concentrate on how this could alter the HbA1c reading. CLINICAL REASONING: HbA1c is a reflection of long-term glucose control, typically representing the average blood glucose level over 2-3 months. The fact that the patient's HbA1c result contradicts his consistently elevated fingerstick blood glucose readings leads me to suspect that some factor may be affecting the HbA1c result. Sickle cell disease is likely relevant, as it alters the properties of red blood cells, which could directly influence HbA1c levels. Due to the sickling and subsequent chronic hemolysis, the lifespan of red blood cells is shortened (10-20 days compared to the normal 120 days. Thus, glucose has less time to bind to hemoglobin (thus forming HbA1c) which may result in a falsely low HbA1c. CONCLUDING: Considering that HbA1c is a cumulative measure of glucose control over the lifespan of red blood cells, its precision could be uncertain in cases where red blood cells have a shortened lifespan, such as in sickle cell disease. This phenomenon might also clarify why this patient's HbA1c reading appears better than what his average home blood glucose concentrations would suggest. RESPONSE: Therefore, likely due to sickle cell disease, the patient's HbA1c is lower than it should be given his reported blood glucose concentrations (Answer: E).

**AI_E:** RESTATING THE QUESTION: The query seeks to decipher why a young male patient with known sickle cell disease and type 1 diabetes exhibits a discrepancy between his home-monitored blood glucose levels, which are relatively quite high (values mentioned indicate poor glycemic control), and his hemoglobin A1c (HbA1c), which at 5.4% denotes good glycemic control. Furthermore, the patient's compliance with his insulin regimen and dietary measures has been questionable. HbA1c is a measure of the average plasma glucose concentration over prolonged periods (approximately 3 months) and provides a robust reflection of long-term glycemic control. PLANNING: The assumptions that could explain this situation include possibilities of inaccurate glucometer readings, better glycemic control than observed, clinical laboratory error in HbA1c measurement, an underlying iron-deficiency anemia, or a confounding effect of the patient's sickle cell disease on his HbA1c levels. Among these, the presence of sickle cell disease, a known hemoglobinopathy, stands as a viable clinical priority given its potential to substantially interfere with HbA1c test results. CLINICAL REASONING: Sickle cell disease, a genetic disorder, causes red blood cells (RBCs) to assume a sickle shape that is prone to hemolysis, consequently reducing their lifespan. This shorter lifespan (10–20 days compared to 120 days for normal RBCs) limits glucose exposure time and artificially lowers HbA1c values independent of the actual average glucose levels. Furthermore, since HbA1c is a form of hemoglobin specifically connected with glucose, and the patient's sickle cell disease specifically alters hemoglobin, it stands to reason that the patient's HbA1c level could be inaccurately low. In this case, standard HbA1c testing may not truly reflect the patient's glycemic control, leading to the observed discrepancy. CONCLUDING: Through the process of elimination and focusing on clinical relevance, the most probable cause for the apparent inconsistency in the patient's glucose and HbA1c values can be attributed to the alteration of hemoglobin structure and RBC lifespan caused by his sickle cell disease, rather than glucometer error, better-than-observed control, lab error, or iron-deficiency anemia. Specifically, the sickle cell disease leads to an underestimation of the HbA1c level. RESPONSE: (E) His sickle cell disease is affecting his hemoglobin A1c.

**AI_D:** RESTATING THE QUESTION: The primary concern here revolves around the discordance observed between a young boy's elevated home glucose readings and his relatively normal Hemoglobin A1c levels. The boy has a history of sickle cell disease and Type 1 diabetes. Hemoglobin A1c, for context, is an important marker in managing diabetes as it reflects the average blood glucose concentration over 2-3 months, while fingerstick glucose measurements provide spot-checks of current glucose levels. PLANNING: Several assumptions can be made, each corresponding with one of the answer choices. (A suggests the boy has iron deficiency anemia, reducing his HbA1c; (B implies his glucose control is better than his diary suggests; (C contends his glucometer is inaccurate and overestimating his glucose levels; (D proposes that the HbA1c lab result is erroneous and should be retested; (E posits that his sickle cell disease is falsely lowering his HbA1c level). Each assumption has a basis in a different pathophysiological mechanism, and we have to ascertain the likelihood of each while considering the boy's specific clinical scenario. CLINICAL REASONING: Regarding assumption A, iron deficiency anemia can technically lower the HbA1c level, since it slows down the glycation of hemoglobin. However, there are no signs of anemia in the boy's presented clinical picture. Assumption B- improved glucose control- is unlikely given the information about his non-compliance with the treatment regimen which would theoretically lead to higher glucose levels. Assumption C, while plausible, is hard to confirm without additional information like glucometer calibration checks. Assumption D, a lab error, cannot be completely ruled out. However, lab error should never be a first consideration unless there is strong evidence to support it. Then we have assumption E which concerns the effect of sickle cell disease on this boy's HbA1c levels. Sickle cell disease can indeed lower HbA1c values as sickled red blood cells have shorter lifespans and there's less time for hemoglobin to become glycated. CONCLUDING: After careful deliberation, we prioritize the assumption that takes into account the patient's significant history of sickle cell disease. This unique attribute of the patient's health history likely acts as the primary confounding factor between his home fingerstick glucose measures and HbA1c values. RESPONSE: So, in conclusion, the most probable explanation for the discrepancy between the patient's home fingerstick blood glucose concentrations and his hemoglobin A1c is (E) His sickle cell disease is affecting his hemoglobin A1c.

**AI_B:** RESTATING THE QUESTION: A young male patient, who has both sickle cell disease and type 1 diabetes mellitus, exhibits a discrepancy between his home fingerstick blood glucose readings and his hemoglobin A1c result. While his home glucose readings are relatively high, his recent hemoglobin A1c reading was 5.4%, which is considered normal and indicative of good glucose control. We are asked to identify the most likely explanation for this discrepancy. The options include factors such as iron deficiency anemia, more effective glucose control than recorded, glucometer errors, a laboratory mistake in the hemoglobin A1c reading, or the influence of his sickle cell disease on the hemoglobin A1c result. PLANNING: One assumption might be that the patient's home urinalysis readings are accurate and his glucose control has indeed been poor. However, counter to this, the patient's hemoglobin A1c indicates good control. This discrepancy could potentially be due to discrepancies in the data collection equipment, variances due to the patient's concurrent sickle cell disease, or an error in the laboratory that processed the A1c result. The alternative assumption could be that the home reading equipment (glucometer) is faulty, producing falsely high readings. Finally, it's also possible the patient's daily glucose control is better than what's recorded in his diabetic diary considering he admits to checking his glucose concentration infrequently. CLINICAL REASONING: If there were an error in the laboratory analysis of the patient's hemoglobin A1c or if the patient had iron deficiency anemia, we would most likely see other clinical manifestations or lab findings, not only a discrepancy between glucose control measurements. Similarly, if the patient's glucose control is indeed better than recorded, we would expect the patients and family to provide some evidence of this. Therefore, the discrepancy might be attributed to other factors, such as the patient's concurrent sickle cell disease or possible glucometer errors. Sickle cell disease (SCD) is a genetic disorder affecting the structure of red blood cells. It can potentially alter the lifespan and turnover of red blood cells, subsequently affecting the accumulation of glycosylated hemoglobin (HbA1c). Specifically, SCD patients might have shortened red blood cell survival, thus allowing less time for hemoglobin to become glycated and making HbA1c values an unreliable measure of long-term glycemic control. CONCLUDING: In this case, the most likely explanation for the discrepancy between the patient's home blood glucose readings and his A1c results is the patient's sickle cell disease affecting his HbA1c values. RESPONSE: The answer is (E) His sickle cell disease is affecting his hemoglobin A1c.

**AI_C:** (1) RESTATING THE QUESTION: The question at hand raises a concern about a discrepancy between a 12-year-old boy's hemoglobin A1c level and his home fingerstick blood glucose concentrations. The hemoglobin A1c test is used to measure the average blood glucose level over the past 2-3 months, while home fingerstick glucose test informs about the immediate glucose level. The boy, suffering from Type 1 diabetes mellitus and sickle cell disease, has a history of inconsistency in adhering to his insulin and dietary protocols. Despite high glucose levels, his HbA1c level is at 5.4%, lower than one would expect considering his glucose levels. (2) PLANNING: Several assumptions stem from this problem. First, his glucometer might be incorrect, reading falsely high glucose levels. Alternatively, his daily glucose control could be better than what is recorded. Another assumption is that lab error might be causing inaccurately low HbA1c results. Other possibilities are that he has iron deficiency anemia, or his sickle cell disease may be affecting his HbA1c levels. Prioritizing these claims, the most pertinent one can be the influence of sickle cell disease on his hemoglobin A1c levels. (3) CLINICAL REASONING: Hemoglobin A1c (HbA1c) is a marker used to determine the average glucose level in the blood over a period of 2-3 months. Considering that the patient's home fingerstick glucose levels are consistently elevated (140–200 mg/dL) and he is partially noncompliant with his insulin and dietary regimens, one would expect a correspondingly high HbA1c value. However, his HbA1c is relatively low (5.4%), which renders a clinical discrepancy. This suggests that factors other than simple glucose control may be influencing the HbA1c values. In sickle cell disease, red blood cells, that carry hemoglobin molecules, have a significantly reduced lifespan due to their sickling and subsequent removal from circulation. This turnover interferes with the glycation process of hemoglobin, leading to an artificially lowered HbA1c level. In essence, the glycation, the process by which glucose molecules attach to hemoglobin, may not have enough time to progress fully due to the faster red cell turnover. (4) CONCLUDING: Thus, through a step-by-step explanation and medical reasoning process, it is plausible to consider that the considerable discrepancy between the patient's home fingerstick blood glucose levels and the hemoglobin A1c might be a consequence of his sickle cell disease. (5) RESPONSE: Therefore, it can be concluded that the most likely explanation for the discrepancy between the patient's home fingerstick blood glucose concentrations and his hemoglobin A1c is (E) His sickle cell disease is affecting his hemoglobin A1c.

**Facilitator** The consensus statement synthesizes the responses of the five AI opinions as follows: The question revolves around a 12-year-old boy with both sickle cell disease and type 1 diabetes mellitus, who exhibits a discrepancy between his home fingerstick blood glucose readings and his hemoglobin A1c (HbA1c) level. Despite his home glucose readings being relatively high, indicative of poor glycemic control, his HbA1c level is 5.4%, which is considered normal and suggests good long-term glucose control. The task is to identify the most likely explanation for this discrepancy. Several assumptions could account for this discrepancy, including the possibility of anemia, better glucose control than recorded, a problem with the glucometer, a lab error with the HbA1c, or an influence of sickle cell disease. Evaluating each of these assumptions, the boy's existing sickle cell disease prompts us to focus on how this could alter the HbA1c reading. HbA1c is a reflection of long-term glucose control, typically representing the average blood glucose level over 2-3 months. The fact that the patient's HbA1c result contradicts his consistently elevated fingerstick blood glucose readings leads us to suspect that some factor may be affecting the HbA1c result. Sickle cell disease is likely relevant, as it alters the properties of red blood cells, which could directly influence HbA1c levels. Due to the sickling and subsequent chronic hemolysis, the lifespan of red blood cells is shortened (10-20 days compared to the normal 120 days). Thus, glucose has less time to bind to hemoglobin (thus forming HbA1c) which may result in a falsely low HbA1c. Considering that HbA1c is a cumulative measure of glucose control over the lifespan of red blood cells, its accuracy could be compromised in cases where red blood cells have a shortened lifespan, such as in sickle cell disease. This phenomenon might also explain why this patient's HbA1c reading appears better than what his average home blood glucose concentrations would suggest. Therefore, the consensus is that the most likely explanation for the discrepancy between the patient's home fingerstick blood glucose concentrations and his hemoglobin A1c is that his sickle cell disease is affecting his hemoglobin A1c (Answer: E).
